# The Relationship Between Kinesiophobic Attitude and Frailty in Older People

**DOI:** 10.1111/ijn.70002

**Published:** 2025-03-16

**Authors:** Fatma Zehra Genç, Naile Bilgili

**Affiliations:** ^1^ Department of Public Health Nursing, Coordination of Project Development and Support Necmettin Erbakan University Konya Türkiye; ^2^ Department of Public Health Nursing, Faculty of Nursing Gazi University Ankara Türkiye

**Keywords:** biologic, frailty, kinesiophobia, movement, psychology

## Abstract

**Aim:**

To investigate the relationship between kinesiophobic attitudes and their causes and frailty in older people.

**Methods:**

This descriptive, relationship‐seeking study was conducted with 302 people aged over 65 years. The data were collected through face‐to‐face interviews between July and September 2023, using a personal information form, the Tampa Scale of Kinesiophobia, the Kinesiophobia Causes Scale (KCS) and the Edmonton Frail Scale (EFS). The data were analysed using Pearson's correlation test, linear regression and binary logistic regression.

**Results:**

A total of 92.7% of older adults experienced high levels of kinesiophobia, while 80.5% presented various degrees of frailty. Most people's kinesiophobia is caused by psychological factors. There is a positive and significant correlation between kinesiophobia and frailty, as well as between the causes of kinesiophobia and frailty. The linear regression model showed that age, sex, physical activity, pain score, kinesiophobic attitudes and causes explained 52.1% of the variation in the EFS score. The binary logistic regression model, based on the frailty categorical variable (frail vs. non‐frail), found that age, sex, physical activity, pain score and kinesiophobic attitudes accounted for 49.0% of the variation in the EFS score.

**Conclusions:**

Kinesiophobic attitudes and causes are important risk factors for frailty and can predict an individual's frailty state.

**Summary**

**What is already known about this topic?**

- Frailty, which is encountered in older people, is a geriatric syndrome that causes physiological, psychological and cognitive impairment.
- Both biologic and psychosocial factors cause a lack of physical activity and kinesiophobia.
- Kinesiophobia develops in older people due to many causes and can cause them to have difficulty performing functional activities.

**What this paper adds?**

- There is a positive and significant correlation between kinesiophobia and frailty, as well as between the causes of kinesiophobia and frailty.
- Frailty is associated with variables such as age, female sex, lack of physical activity, kinesiophobic attitudes and causes.

**The implications of this study**

- It is crucial to regularly assess kinesiophobia and its causes to detect frailty in older adults.
- Kinesiophobic attitudes and causes are important risk factors for frailty and can predict an individual's frailty state.

## 1 Introduction

Kinesiophobia is defined as an excessive and irrational fear of physical movement and activity resulting from a feeling of vulnerability due to the avoidance of painful injury or re‐injury (Kori et al. 1990). Problems related to kinesiophobia, the fear of movement (Vlaeyen et al. 1995), are often associated with chronic pain and are among the topics that have been increasingly explored in the medical literature in recent years (Asiri et al. 2021; Luque‐Suarez et al. 2019). A review of the literature shows that kinesiophobia scores in older adults vary between 36.5 and 46.9, and kinesiophobic attitudes are high (Asiri et al. 2021; Genç and Bi̇lgi̇li̇ 2023; Knapik et al. 2019; Sertel et al. 2021; Şevgi̇n and Alpteki̇n 2020; Uchida et al. 2020). An important risk factor for kinesiophobia in older people is problems related to the musculoskeletal system (Güzel et al. 2021). In a systematic review, it was determined that high levels of kinesiophobia were associated with low quality of life and disability in people with musculoskeletal pain (Luque‐Suarez et al. 2019). Ishak et al. (2017) found a significant relationship between kinesiophobia and mobility and balance, and that kinesiophobic attitude was an important predictor of mobility and balance. Another study found that most participants had superficial kinesiophobia, low self‐efficacy and moderate pain intensity, and that gender, self‐efficacy and pain intensity were related to kinesiophobia. In another study, the prevalence of kinesiophobia in older people with knee osteoarthritis was found to be 57.89%, and marital status, educational status, pain level, social support and pain score, and self‐perception burden score were found to be significant determinants of kinesiophobia (Tan et al. 2022). It was shown that kinesiophobia was at a high level in 76% of older people with coronary artery disease (Knapik et al. 2019). In a study based in a nursing home, it was reported that lower extremity pain was much higher in people with chronic pain, which could significantly affect physical activity, and these people experienced a significant phobia of movement. The study also stated that the presence of chronic pain could significantly affect social participation, functional level and quality of life in older people who had problems with physical activity levels, fear of falling and movement phobia due to advancing age (Sertel et al. 2021). Erden and Güner (2018) stated that kinesiophobia affected physical parameters and negatively affected anxiety, depression and quality of life.

Kinesiophobia, which develops in older people due to many causes, can cause them to have difficulty in performing functional activities, difficulty in participating in social life and a decrease in quality of life (Courbalay et al. 2021; Güzel et al. 2021). It is important to evaluate the condition of kinesiophobia, which greatly affects the lives of older people and is characterized by biologic and psychological conditions (Çayir et al. 2020; Trocoli and Botelho 2016). Studies conducted on older people with a variety of health issues indicate that kinesiophobia is related to balance and mobility and limits physical activity, reduces functional skills and participation and causes problems with socialization (Genç and Bi̇lgi̇li̇ 2023; Güzel et al. 2021; Goubran et al. 2024). Both biologic and psychosocial factors cause a lack of physical activity and fear of movement. Saulicz et al. (2016) found that kinesiophobia was at the lowest level in both biologic and psychological domains in men and women who were physically active in their youth. Another study found a significant relationship between kinesiophobia and depression and some sub‐dimensions of quality of life (sleep, social isolation and emotional reactions) (Bağlan Yentür et al. 2019). As biological causes of kinesiophobia, while it is considered morphological, individual needs for stimulation, energy resources, the power of biological drives and psychological reasons are self‐acceptance, self‐evaluation of motor predispositions and body care have been discussed (Knapik et al. 2011; Kocjan and Knapik 2014). Considering both the biologic and psychological causes of kinesiophobia, it is predicted that the state of vulnerability will further refine the kinesiophobic attitude of individuals.

Frailty, which is frequently encountered in older people, is a geriatric syndrome that causes physiologic, psychological and cognitive impairment (Alpalhão et al. 2022). When examining the prevalence of frailty, Alqahtani et al. (2022) found that frailty in Middle Eastern countries, including Turkey, was 35%; O'Caoimh et al. (2021) found that frailty was 12%–24%; Akın et al. (2015) found that frailty in Turkey was 10.2%–48.5%; To et al. (2022) found that frailty in Asian countries, including Turkey, was 20.5%. Kwak and Thompson (2021) stated that frailty was preventable, and therefore it was important to determine the frailty rate and factors that affected frailty. In this context, efforts should be made to detect fragility in societies at an early stage and identify and improve risk factors. Although studies have shown that age and sex influence frailty (Qiu et al. 2024; To et al. 2022), no literature was found on kinesiophobic attitudes and causes. In a kinesiophobic attitude, avoidance of fear can lead to physical inactivity and disability, which can lead to frailty (Alpalhão et al. 2022; Barğı and Koku 2022). As a result of kinesiophobic attitudes, older people may become frail and experience both physical and psychosocial weaknesses. In this context, the causes of kinesiophobia and kinesiophobia in older people should be considered together with frailty. Although kinesiophobia and its causes in older people have been discussed in different disease groups in the literature, no studies examining its relationship with frailty have been found. This study aimed to determine the relationship between kinesiophobic attitude and its causes and frailty in older people.

*Research questions*

- What level of kinesiophobic attitudes is present in older people?
- What are the causes of kinesiophobic attitudes in older people?
- What level of frailty do older people have?
- Is there a relationship between kinesiophobia and frailty in older adults?

## 2 Methods

### 2.1 Design

This study was conducted using a descriptive, relationship‐seeking approach and was reported in accordance with the STROBE checklist.

#### 2.1.1 Population and Sample of the Research

The study group consisted of people aged 65 years and over who were admitted to a family health centre in Turkey between July and September 2023. Three hundred two volunteer older people who had no problems with verbal communication constituted the sample of this research. The family health centre where the study was conducted had a population of 1340 individuals aged 65 years and over. Sample size calculations, using a formula for known populations, determined that with an acceptable error margin of 5%, a confidence level of 95% and a population of 1340, the minimum required sample size was 299. The study ultimately included 302 older adults, chosen through haphazard sampling, a non‐probability sampling method. Post‐hoc analysis using the G*Power 3.1.9.7 software showed that with a regression coefficient of 0.438 from our study, the effect size was 0.779. When the effect size of 0.779, a type I error of 0.05 and a sample size of 302 were entered into the programme, the study's power was determined to be 100%.

### 2.2 Criteria for Inclusion in the Research

This study included individuals aged 65 years and over who had no severe limb, vision or hearing impairments, had not undergone surgery in the past 3 months and were in good mental health, as indicated by a Mini‐Mental State Examination score of 24 or higher.

### 2.3 Dependent and Independent Variables

The dependent variable of this research is frailty; the independent variables are age, sex, physical activity, pain score, kinesiophobic attitudes and cause.

### 2.4 Collection of Data

Data were collected between July and September 2023 through face‐to‐face interviews. Data were collected using a personal information form, the Visual Analogue Scale (VAS), the Standardized Mini‐Mental State Examination (SMMSE), the Tampa Scale of Kinesiophobia (TSK), the Kinesiophobia Causes Scale (KCS) and the Edmonton Frail Scale (EFS). Data collection took approximately 15–18 min.

### 2.5 Data Collection Tools

#### 2.5.1 Personal Information Form

This was prepared by the researchers in line with the literature (Alpalhão et al. 2022; Ayrancı and Dundar 2021; John et al. 2022; Sayilan et al. 2022; Sertel et al. 2021; Telatar et al. 2020).

#### 2.5.2 Visual Analogue Scale (VAS)

The VAS was developed to measure the intensity of pain in individuals. The VAS is a safe, easy‐to‐use measurement tool that is used to convert some values that cannot be measured numerically into numerical values and has been widely accepted in the world literature for many years. A low score indicates that the individual's pain intensity is low or moderate, and a high score indicates that the pain intensity is high or severe. Participants rated pain intensity on a scale from 1 (*no pain at all*) to 10 (*excruciating pain*) (Wewers and Lowe 1990).

#### 2.5.3 Standardized Mini‐Mental State Examination (SMMSE)

This scale was used to assess participants' cognitive status and determine whether they would be included in the study. A maximum of 30 points can be obtained from the test, and higher scores indicate better mental status. A score of 24 or more indicates normal mental function (Folstein 1975; Güngen et al. 2002).

#### 2.5.4 The Tampa Scale of Kinesiophobia (TSK)

The scale is a 4‐point Likert‐type scale containing 17 questions, which measure injury/re‐injury and fear avoidance in work‐related movements. Vlaeyen et al. (1995) published the original scale with the permission of the researchers who developed it. Yılmaz et al. (2011) performed the Turkish validity and reliability study of the scale. The total score is calculated after the 4th, 8th, 12th and 16th items of the scale are reversed, and the total score varies between 17 and 68. Increased total scores on the scale mean that kinesiophobia also increases in the individuals. It has been stated that a score of 37 or above on the scale indicates a high degree of kinesiophobia. Yılmaz et al. (2011) found the reliability of the scale to be 0.80. The reliability coefficient of the scale in our study was 0.84.

#### 2.5.5 The Kinesiophobia Causes Scale (KCS)

This scale, consisting of 20 five‐point Likert‐type questions, enables the determination of biologic and psychological causes of kinesiophobia. It is important to determine the causes of motor inactivity and fear of movement. The scale consists of two subscales, biologic and psychological. The parameters that constitute the biologic sub‐dimensions are morphologic, individual need for stimulation, energetic substrates and power of biologic drives. The parameters that constitute the psychological sub‐dimensions are self‐acceptance, self‐assessment of motor predispositions and body care (Knapik et al. 2011; Kocjan and Knapik 2014). Çayir et al. (2020) performed the Turkish validity and reliability study of the scale, and the reliability coefficient was determined as 0.86. The reliability coefficient of the scale in our study was 0.90.

#### 2.5.6 Edmonton Frail Scale (EFS)

The scale consists of nine frailty dimensions and a total of 11 items, which are considered determinants in the assessment of frailty in older people (Rolfson et al. 2006). Frailty dimensions are considered as ‘cognition, general health status, functional independence, social support, medication use, nutrition, mood, continence, function performance’. The Turkish validity and reliability study of the scale was performed by Aygör et al. (2018). The score that can be obtained from the scale varies between 0 and 17. Increasing the score increases the degree of frailty. If the total score is 0–4, it is categorized as not frail; 5–6, vulnerable; 7–8, mild frailty; 9–10, moderate frailty; and 11 and above, severe frailty (Aygör et al. 2018; Rolfson et al. 2006). The reliability coefficient of the scale was determined as 0.75. The reliability coefficient in our study was 0.61.

### 2.6 Evaluation of Data

The Statistical Package for the Social Sciences Ver. 22.0 software package was used for data analysis. *p*‐values of < 0.05 were accepted as statistically significant, the margin of error as 0.05 and confidence intervals as 95%. For the analysis of personal characteristics, categorical variables are presented as frequency and percentage, and continuous variables as mean and standard deviation. The normality of data distribution was evaluated according to skewness and kurtosis values, and the range of −1.5 to +1.5 was taken into account (Tabachnick and Fidell 2013). Pearson correlation analysis was performed to examine the relationship between kinesiophobic attitude and kinesiophobia causes and frailty. Linear regression analysis was used to determine the relationship between kinesiophobic attitude and its causes and frailty. In addition, frailty status was considered as a binary categorical variable (a score of 4 or less is not frail, a score of 5 or more is frail), and the relationship between frailty and kinesiophobic attitudes and causes was examined using binary logistic regression.

### 2.7 Ethical Considerations

Prior to commencing the research, approval was obtained from the University Ethics Commission (Research Code No: 2023‐540, April 20, 2023), and institutional permission was granted by the Provincial Health Directorate. Additionally, consent was obtained from the participants after providing them with all the relevant information. The scale developers also gave permission via email.

## 3 Results

### 3.1 Descriptive Results

The participants had an average age of 72.3 years and an average pain score of 5.09. The pain locations included the knee (31.1%), general body (24.8%), waist (16.9%), head (10.6%) and arm/shoulder (6%) regions. Of the participants, 59.9% were women, 78.5% were married, 60.6% were primary/secondary school graduates and 75.2% were non‐smokers. According to recent research, almost 70% of the older people use assistive devices, such as canes or walkers, to help them move around. On the other hand, only 25.8% exercise regularly by engaging in activities such as walking or jogging for at least 30 min per day on at least 5 days of the week, which leads to sweating and accelerated breathing (Table 1).

**TABLE 1:** Characteristics of the older people.

	*n*	%
Sex		
Female	181	59.9
Male	121	40.1
Marital status		
Married	237	78.5
Single	65	21.5
Education status		
Illiterate/literate	76	25.2
Primary/secondary school	183	60.6
High school/bachelor	43	14.2
Smoking		
No	227	75.2
Yes	75	24.8
Use of assistive devices to act		
No	210	69.5
Yes	92	30.5
Doing physical activity		
No	224	74.2
Yes	78	25.8

	Mean ± SD	Median (min–max)
Age	72.3 ± 6.08	70.5 (65–88)
Pain score	5.09 ± 2.17	5 (1–9)
Tampa Scale of Kinesiophobia (TSK)	48.21 ± 7.68	49 (24–60)

	*n*	%
High (TSK ≥ 37)	280	92.7
Low (TSK < 37)	22	7.3

	Mean ± SD	Median (min–max)
Kinesiophopia Causes Scale	3.37 ± 0.59	3.4 (1.6–4.5)
Biological sub‐dimension	3.32 ± 0.62	3.4 (1.8–4.6)
Morphological	3.34 ± 1.29	4 (1–5)
The individual need for stimulation	2.66 ± 0.42	2.7 (1–3.7)
Energy resources	3.46 ± 1.13	3.8 (1–5)
The power of biological drives	3.80 ± 0.70	4 (2–5)
Psychological sub‐dimension	3.43 ± 0.72	3.6 (1.2–4.9)
Self‐acceptance	3.62 ± 1.03	4 (1–5)
Self‐assessment of motor predispositions	3.01 ± 0.94	3 (1–5)
Body care	3.67 ± 0.78	4 (1.3–5)
Edmonton Frail Scale	7.13 ± 2.94	7 (0–13)

	*n*	%
Not frail	59	19.5
Vulnerable	63	20.9
Mild frailty	85	28.1
Moderate frailty	58	19.2
Severe frailty	37	12.3

The participants had an average TSK score of 48.21, with 92.7% exhibiting high kinesiophobia (TSK ≥ 37). It was determined that kinesiophobia was mostly caused by psychological factors (3.43). The participants had an average EFS score of 7.13, with only 19.5% not considered frail. Table 1 shows that 80.5% of participants had varying levels of frailty, with 20.9% classified as vulnerable, 28.1% as mildly frail, 19.2% as moderately frail and 12.3% as severely frail.

### 3.2 Statistical Results

Pearson correlation analysis was used to examine the relationship between the scale scores of the older adults (Table 2). The positive and significant relationship between kinesiophobia (*r* = 0.616, *p* < 0.01) and frailty, and the relationship between causes of kinesiophobia and frailty (*r* = 0.615, *p* < 0.01). Upon examining the causes of kinesiophobia, the biologic subdimension (*r* = 0.616, *p* < 0.01) was found to have a higher significant relationship with frailty than the psychological subdimension (*r* = 0.483, *p* < 0.01), as shown in Table 2.

**TABLE 2:** Relationship between scale scores of older people.

	1	2	3	4	5	6	7	8	9
1. Age	1.000								
2. Sex	0.002	1.000							
3. Doing physical activity	0.232*	0.150*	1.000						
4. Pain score	0.116**	0.276*	0.376*	1.000					
5. Tampa Scale of Kinesiophobia	0.296*	0.174*	0.544*	0.453*	1.000				
6. Kinesiophopia Causes Scale	0.312*	0.203*	0.521*	0.323*	0.739*	1.000			
7. Biological sub‐dimension	0.311*	0.252*	0.545*	0.371*	0.757*	0.869*	1.000		
8. Psychological sub‐dimension	0.248*	0.117**	0.391*	0.213*	0.568*	0.903*	0.571*	1.000	
9. Edmonton Frail Scale	0.423*	0.232*	0.521*	0.399*	0.616*	0.615*	0.616*	0.483*	1.000

* Correlation is significant at the 0.01 level (2‐tailed).

** Correlation is significant at the 0.05 level (2‐tailed).

Linear regression analysis was performed to investigate the relationship between kinesiophobia and frailty (see Table 3). Statistical analysis showed that the regression model developed to predict frailty status was significant (*p* = 0.047). Increasing age (*p* < 0.001), being female (*p* = 0.047), not doing physical activity (*p* = 0.001) and increasing pain score (*p* = 0.022) increase frailty. A one‐unit increase in the TSK score increases the frailty score by 0.191 units (*p* = 0.005). A one‐unit increase in biological causes of kinesiophobia increases the frailty score by 0.181 units (*p* = 0.007). A one‐unit increase in the psychological causes of the kinesiophobia scale score increased the vulnerability score by 0.117 units (*p* = 0.022). Table 3 shows that 52.1% of the variation in the EFS score was explained by age, sex, physical activity, pain score, kinesiophobic attitudes and causes.

**TABLE 3:** Linear regression analysis according to frail score.

	β^1^	S.E.	β^2^	*t*	*p*	Zero‐order	Partial	VIF
(Constant)	−10.785	1.489		−7.242	< 0.001			
Age	0.111	0.021	0.230	5.406	< 0.001	0.423	0.301	1.133
Sex (female)	0.507	0.254	0.085	1.997	0.047	0.232	0.116	1.129
Doing physical activity (no)	1.118	0.335	0.167	3.337	0.001	0.521	0.191	1.566
Pain score	0.145	0.063	0.107	2.295	0.022	0.399	0.133	1.372
Tampa Scale of Kinesiophobia	0.073	0.026	0.191	2.838	0.005	0.616	0.163	2.841
Biological sub‐dimension	0.858	0.314	0.181	2.735	0.007	0.616	0.158	2.766
Psychological sub‐dimension	0.480	0.208	0.117	2.309	0.022	0.483	0.133	1.609

*Note:* F = 3.990; *p* = 0.047; Adj. *R* ^2^ = 0.521; Durbin Watson = 1.984.

Abbreviations: β^1^ = unstandardized coefficients, β^2^ = standardized coefficients.

Frailty status was categorized as either frail or non‐frail, and binary logistic regression was used to examine the relationship between frailty and kinesiophobic attitudes and causes (see Table 4). The model developed to predict fragility (binary) was statistically significant (*p* < 0.001). According to the binary logistic regression model based on the frailty binary categorical variable, which includes frail and non‐frail, 49.0% of the variation in EFS score was accounted for by age, sex, physical activity, pain score and kinesiophobic attitudes. Increasing an individual's TSK score by one unit increased the risk of being frail by 1.128 times (*p* < 0.001). When age, sex, no physical activity, pain score, TSK and the biologic subdimension of the KCS variables are added to the established model one by one, it is seen that all variables separately influence frailty. However, when all variables were added to the model together, it was found that the variables age, sex, no physical activity, pain score and TSK score were effective.

**TABLE 4:** Binary logistic regression analysis according to frail status.

	Univariate		Multivariate (Wald)*	
	OR (95% CI)	*p*	OR (95% CI)	*p*
Age	1.144 (1.073–1.220)	< 0.001	1.162 (1.065–1.268)	0.001
Sex (female)	0.287 (0.158–0.520)	< 0.001	0.259 (0.118–0.567)	0.001
Doing physical activity (no)	0.098 (0.052–0.186)	< 0.001	0.368 (0.163–0.831)	0.016
Pain score	1.605 (1.372–1.879)	< 0.001	1.231 (1.014–1.494)	0.035
Tampa Scale of Kinesiophobia	1.204 (1.143–1.269)	< 0.001	1.128 (1.059–1.202)	< 0.001
Biological sub‐dimension	8.570 (4.696–15.638)	< 0.001		
Psychological sub‐dimension	3.643 (2.367–5.606)	< 0.001		

*Note:* Nagelkerke *R* ^2^ = 0.490, accurary = 80.5%.

Abbreviations: CI = confidence interval, OR = odds ratio.

## 4 Discussion

Kinesiophobia can cause older adults to avoid physical activity, leading to decreased mobility and frailty (Barğı and Koku 2022; Ishak et al. 2017; Malouka et al. 2023). Despite experiencing pain and fear, it may not always be advisable to suggest that individuals should avoid activities or movements that cause discomfort. Doing so can lead to further limitations in physical activity, which can cause muscles to weaken and result in disuse, thereby increasing the risk of frailty (Ishak et al. 2017). In this context, it is crucial to understand the relationship between kinesiophobia and frailty in older adults, identify its causes and overcome it. The relationship between kinesiophobia and frailty in older people is highlighted in this study, emphasizing the importance of kinesiophobic attitudes and causes as determinants of frailty.

In our study, it was found that 25.8% of older adults engaged in moderate‐intensity physical activity for at least 5 days a week, similar to Genç and Bi̇lgi̇li̇'s (2023) study. The participants reported a pain score of 5.09, which indicates moderate pain. According to a study conducted by John et al. (2022), 58% of adults experienced moderate pain. In another study by Felício et al. (2016), it was found that older women experienced moderate pain with an average pain score of 4.7 on the numerical VAS. Our study found that almost 60% of the participants were women, and their pain scores were similar. According to Genç and Bi̇lgi̇li̇ (2023), older people living in nursing homes had an average pain score of 2.16. Factors such as age, sex, chronic disease and living environment (nursing home vs. community) may contribute to differences in pain levels.

The study group reported pain in the knee (31.1%), general body (24.8%), waist (16.9%), head (10.6%) and arm/shoulder (6%). According to several studies, older people tend to experience various types of pain. Uchida et al. (2020) found that back pain was reported by 55% of older people, knee pain by 50% and shoulder pain by 25.3%. Meanwhile, Sertel et al. (2021) determined that 47.2% of older people had knee pain, 37.4% had pain in the cervical, lumbar or thoracic spine and 9.8% had shoulder pain. Additionally, Genç and Bi̇lgi̇li̇ (2023) discovered that low back pain was reported by 92% of older people. Lastly, Ishak et al. (2017) specifically studied those who reported low back pain. In all the studies, it is seen that the areas where pain is frequently seen are similar and severe in the knee, waist/back and shoulder (Genç and Bi̇lgi̇li̇ 2023; Ishak et al. 2017; Sertel et al. 2021; Uchida et al. 2020). Ahangari and Abdolrahmani (2020) stated that there was a correlation between chronic pain, low back pain, neck pain and falls, and high kinesiophobia. In our study, we found that the pain score and kinesiophobia were positively related (*r* = 0.453, *p* < 0.01) and that the pain score was also positively related to an individual's frailty (*r* = 0.399, *p* < 0.01). It is crucial to identify painful areas in older people and determine the degree of pain to prevent kinesiophobic attitudes and frailty. These problems can lead to a decline in functional capacity, reduced performance in daily living activities, increased addiction and a lower quality of life.

In our study, we used the TSK‐17 form, which was validated and found reliable in Turkish. Knapik et al. (2019) showed that the TSK score was 43.02, Şevgi̇n and Alpteki̇n (2020) as 42.73 and 43.13, Uchida et al. (2020) as 36.5, Asiri et al. (2021) as 46.9, Sertel et al. (2021) as 44.42 and Genç and Bi̇lgi̇li̇ (2023) as 43.72. In studies with high kinetophobic attitudes (Asiri et al. 2021; Sertel et al. 2021), it is seen that the sample consists of people experiencing chronic pain. In our study, we found that the chronic pain score was moderate and the average TSK score was 48.21. Using the TSK‐11 form, which was a 4‐point Likert‐type scale, Ishak et al. (2017) found that people with low back pain had an average TSK‐11 score of 29.67 and a moderate level of kinesiophobia, and Larsson et al. (2016) determined that the TSK‐11 score was 22.8. Knapik et al. (2019) found that 76.3% of older people with coronary artery disease had high kinesiophobia, and in our study, 92.7% of people were found to have high kinesiophobia. The high level of kinesiophobic attitudes in our study may be related to variables such as high pain scores and the fact that most older people do not engage in physical activity.

Kinesiophobia, which is the fear of movement, can be caused by both biologic and psychological factors. Researchers have developed a useful tool to identify and measure these factors in individuals and societies (Çayir et al. 2020; Knapik et al. 2011; Kocjan and Knapik 2014). In a study conducted by Genç and Bi̇lgi̇li̇ (2023), the average KCS score of older adults was found to be 3.12, with the biologic domain score at 3.14 and the psychological domain score at 3.11. On the other hand, Çayir et al. (2020) discovered that the KCS total score was 2.54, with the biologic domain score at 2.62 and the psychological domain score at 2.46. Saulicz et al. (Saulicz et al. 2016) revealed that kinesiophobia was mostly caused by psychological factors. Our study's findings align with Saulicz et al. (2016) in terms of the predominance of psychological factors but differ from those of Çayir et al. (2020) and Genç and Bi̇lgi̇li̇ (2023). Our results indicate that the primary cause of kinesiophobia is psychological with a score of 3.43. When we looked at its effect on frailty, we found that the biologic causes of kinesiophobia had a greater effect on frailty (Tables 3 and 4). Looking at the biologic causes of kinesiophobia, the sub‐dimensions and items of the scale are similar to the items and sub‐dimensions of the Frailty Scale. In this respect, it is expected that the biologic causes of kinesiophobia will have a greater impact on frailty.

As people age, their physical and cognitive health may lead to fear of movement, balance issues, muscle weakness and frailty. Frailty is a significant geriatric syndrome, affecting a large percentage of older people. According to meta‐analyses, the incidence of frailty and prefrailty among older adults is around 58% and 63.2%, respectively (O'Caoimh et al. 2021; Veronese et al. 2021). However, some studies reported even higher rates of frailty: 82.5% (Eyigor et al. 2015), 63.2% (Elbi and Özyurt 2021), 60.8% (Aygör et al. 2018) and 73.66% (Akyol Guner 2022). Our study found that 80.5% of older people were frail to varying degrees.

The high frailty rate in our study could be attributed to the similar sociocultural characteristics of the participants (Table 1). Among the factors associated with frailty were sociocultural variables such as age, being female, being a housewife, being inactive, having comorbidities, taking many medications and avoiding going out (Eyigor et al. 2015; O'Caoimh et al. 2021; Qiu et al. 2024). In addition to these variables known in the literature, we also found an association between kinesiophobic attitudes and frailty (Tables 2, 3, 4). In addition to the variables of age, sex, physical activity and pain score, kinesiophobic attitudes and causes should also be considered when assessing an individual's frailty (Tables 3 and 4). Therefore, it is imperative to consider kinesiophobic attitudes and their underlying causes when assessing an individual's frailty.

It is important to note the limitations and strengths of our research. First, the study was conducted in a single centre between July and September 2023, which means our results can only be generalized to this sample. Secondly, the study results were based on the self‐reporting of older people, which may lead to biases. Additionally, because our study was a descriptive and relationship‐seeking study, the ability to establish any causal relationship was limited. Therefore, analytical studies are needed to reveal causal relationships. Although there are limitations, this study is valuable for understanding the link between kinesiophobic attitudes and vulnerability and provides a basis for future research.

## 5 Conclusion

This study found that kinesiophobia is a significant risk factor for frailty in older adults and should be considered because it can predict frailty. It is crucial to regularly assess kinesiophobia and its causes to detect frailty in older adults. Therefore, researchers must approach kinesiophobia from a biopsychosocial perspective to enhance the health and independence of older people. Additionally, the prevalence of frailty is associated with the increase in the size of the older population, making it even more critical to monitor the levels of kinesiophobia and its causes. To minimize the adverse effects of kinesiophobia on individuals, preventive and remedial programmes must closely track kinesiophobia and its causes. Identifying kinesiophobic attitudes, their causes and their relationship to frailty from a prophylactic and therapeutic standpoint may be a necessary and fundamental starting point for any coordinated action.

## Author Contributions

**Fatma Zehra Genç:** conceptualization, methodology, investigation, data curation, formal analysis, writing – original draft. **Naile Bilgili:** conceptualization, methodology, writing – review and editing, supervision.

## Ethics Statement

Prior to commencing the research, approval was obtained from the University Ethics Commission (Research Code No: 2023‐540, April 20, 2023), and institutional permission was granted by the Provincial Health Directorate. Additionally, consent was obtained from the participants after providing them with all the relevant information. The scale developers also gave permission via email.

## Conflicts of Interest

The authors declare no conflicts of interest.

## Data Availability

The findings of this study can be obtained from the relevant corresponding author upon request.
